# The AHR pathway represses TGFβ-SMAD3 signalling and has a potent tumour suppressive role in SHH medulloblastoma

**DOI:** 10.1038/s41598-019-56876-z

**Published:** 2020-01-10

**Authors:** Nemanja Sarić, Matthew Selby, Vijay Ramaswamy, Marcel Kool, Brigitta Stockinger, Christer Hogstrand, Daniel Williamson, Silvia Marino, Michael D. Taylor, Steven C. Clifford, M. Albert Basson

**Affiliations:** 1 Centre for Craniofacial and Regenerative Biology, King’s College London, Floor 27, Guy’s Hospital Tower Wing, London, SE1 9RT UK; 2 Wolfson Childhood Cancer Research Centre, Northern Institute for Cancer Research, Newcastle University, Newcastle-upon-Tyne, NE1 7RU UK; 3 Divisions of Hematology/Oncology and Neurosurgery, The Hospital for Sick Children, Toronto, ON Canada; 4 Departments of Medical Biophysics and Paediatrics, University of Toronto, Toronto, ON Canada; 5 Hopp Children’s Cancer Center (KiTZ), Division of Pediatric Neurooncology, German Cancer Research Center (DKFZ), and German Cancer Consortium (DKTK), Heidelberg, Germany; 6 The Francis Crick Institute, 1 Midland Road, London, NW1 1AT UK; 7 Diabetes & Nutritional Sciences Division, King’s College London, 3.85 Franklin-Wilkins Building, London, SE1 9NH UK; 8 Blizard Institute, Barts and The London School of Medicine and Dentistry, Queen Mary University of London, 4 Newark Street, London, E1 2AT UK; 9 MRC Centre for Neurodevelopmental Disorders, King’s College London, 4th floor, New Hunt’s House, London, SE1 1UL UK

**Keywords:** Cancer genetics, Cancer models, CNS cancer, Neural progenitors, Cancer models

## Abstract

Sonic Hedgehog (SHH) medulloblastomas are brain tumours that arise in the posterior fossa. Cancer-propagating cells (CPCs) provide a reservoir of cells capable of tumour regeneration and relapse post-treatment. Understanding and targeting the mechanisms by which CPCs are maintained and expanded in SHH medulloblastoma could present novel therapeutic opportunities. We identified the aryl hydrocarbon receptor (AHR) pathway as a potent tumour suppressor in a SHH medulloblastoma mouse model. *Ahr*-deficient tumours and CPCs grown *in vitro*, showed elevated activation of the TGFβ mediator, SMAD3. Pharmacological inhibition of the TGFβ/SMAD3 signalling axis was sufficient to inhibit the proliferation and promote the differentiation of *Ahr*-deficient CPCs. Human SHH medulloblastomas with high expression of the AHR repressor (*AHRR*) exhibited a significantly worse prognosis compared to *AHRR*^*low*^ tumours in two independent patient cohorts. Together, these findings suggest that reduced AHR pathway activity promotes SHH medulloblastoma progression, consistent with a tumour suppressive role for AHR. We propose that TGFβ/SMAD3 inhibition may represent an actionable therapeutic approach for a subset of aggressive SHH medulloblastomas characterised by reduced AHR pathway activity.

## Introduction

Medulloblastoma represents one of the most common forms of paediatric, malignant brain tumours accounting for around 20% of all paediatric tumours of the CNS^1^. Typical treatment consists of a combination of chemotherapy, surgical resection and neuraxis irradiation, with a cure rate of approximately 70–75% in children ≥3 years of age^2^. However, survivors of medulloblastoma are left with a host of long-term adverse sequelae, including cognitive deficits, problems with neuroendocrine function and fertility^3,4^. These drawbacks to traditional treatment options necessitate more effective patient stratification strategies based on biomarkers that predict outcome and identify specific molecular medulloblastoma subtypes for personalized, targeted therapies.

Efforts to dissect the molecular underpinnings of medulloblastoma have identified four main subgroups - WNT, Sonic hedgehog (SHH), Group 3 and Group 4 - with distinct transcriptional, DNA methylation and mutational profiles, and different clinical characteristics and outcomes^5–7^. These subgroups are associated with different cells of origin^8^. SHH tumours originate from cerebellar granule cell progenitors (GCPs), and display excessive activation of the SHH signalling pathway^9^. The prognosis of SHH subgroup medulloblastomas is mixed, with some patients doing well and others not, suggesting significant heterogeneity within this subgroup^7^. Indeed, recent work has identified SHH medulloblastoma subtypes with specific molecular characteristics and associated clinical features^5,6^. Independent efforts showed that SHH MBs could be subdivided depending on age and molecular signature. Among these, infant (<3.0–4.0 years at diagnosis) and non-infant SHH subgroups are consistently observed, alongside a subgroup of non-infant tumours associated with a particularly poor prognosis, characterized by amplifications of *MYCN* and *GLI2* and loss of function mutations in *TP53*^5,6^. These molecular signatures have previously been linked to medulloblastomas with metastasis and high rates of post-treatment relapse^6,10,11^. Non-infant SHH tumours without these features are associated with better outcomes (>80% 5-year progression-free survival^5^).

The *Ahr* gene, encoding the aryl hydrocarbon receptor, has been studied extensively in the context of hepatocarcinoma, immune cell development and toxicology^12–14^, where this gene appears to have context-specific roles. In hepatocarcinoma, *Ahr* promotes cell proliferation and tumourigenesis^12^, while it has multiple roles in the immune system^15^. Intriguingly, activation of the AHR pathway by endogenous ligands has been shown to promote brain cancers by the anti-tumour immune response^16^, also suggestive of context-dependent, multi-faceted roles for this pathway in cancer biology. AHR is a bHLH (basic helix-loop-helix) transcription factor which acts as a receptor for endogenous tryptophan metabolites and xenobiotics such as TCDD^17^. Upon ligand binding, AHR translocates to the nucleus, where it forms a complex with formation of a heterodimer with ARNT (Aryl hydrocarbon Receptor Nuclear Translocator). AHR-ARNT heterodimers are recruited to Dioxin Response Elements (DRE) in the genome to regulate gene transcription^18^. An AHR Repressor (AHRR) protein can also dimerise with ARNT to competitively interfere with AHR-ARNT complex formation and inhibit AHR-regulated gene expression^19^.

Sox2+ cancer propagating cells (CPCs), capable of driving tumour initiation and exhibiting enhanced resistance to cytostatic therapy have been identified in SHH medulloblastoma mouse models^20,21^. Lineage tracing experiments of these cells demonstrated their capacity for tumour regeneration following anti-mitotic chemotherapy, suggesting these cells are responsible for tumour relapse^20^. However, the mechanisms governing CPC formation and maintenance in SHH medulloblastomas remain to be fully elucidated. AHR function has been linked to CPC and haematopoietic stem cell maintenance^22,23^. In a recent study, AHR was shown to regulate the balance between quiescence and proliferation in hematopoietic stem cells, with these stem cells becoming less quiescent and more proliferative in *Ahr*-deficient animals^23^.

Previous studies have implicated AHR in cerebellar development and medulloblastoma cell proliferation. *Ahr* deletion in primary cerebellar GCPs^24^ or AHR knock-down in a SHH-associated medulloblastoma cell line^25^ resulted in proliferative deficits. To determine if AHR has a direct role in SHH medulloblastoma *in vivo*, we conditionally deleted the *Ahr* gene in mouse cerebellar GCPs, either alone or in combination with medulloblastoma-initiating *Ptch1* gene deletion. Our analyses of these mice revealed a striking tumour-suppressive role for AHR in mouse SHH medulloblastoma development. We identify a specific role for AHR in regulating the TGFβ-SMAD3 signalling axis in CPCs from these tumours and identify a new role for TGFβ-SMAD3 activity in medulloblastoma CPC differentiation. Examination of the expression of AHR pathway genes in human medulloblastoma cohorts support an important role for the AHR pathway in SHH medulloblastoma biology.

## Results

### AHR modulates primary mouse GCP proliferation and differentiation by repressing TGFβ/SMAD3 signalling

To investigate the role of the AHR pathway in neural progenitor fate in the developing cerebellum, we conditionally deleted the *Ahr* gene from Math1+ GCPs during cerebellar development. In agreement with a previous report^24^, we observed reduced GCP proliferation and enhanced cell cycle exit (as measured by cell Q fraction) (Fig. S1a) of GCPs in *Ahr* conditional knockout *Math1cre; Ahr*^*f/f*^ (*Ahr* cKO) cerebella, compared to control *Ahr*^*f/f*^ cerebella (Fig. S1b,c). The phenotype was particularly prominent in anterior lobules I/II, III, V and VI (Fig. S1d,e). This effect was not observed in the posterior lobules IX/X, which is attributable to lack of Cre activity within these lobules, as described previously^26^.

**Figure 1:**
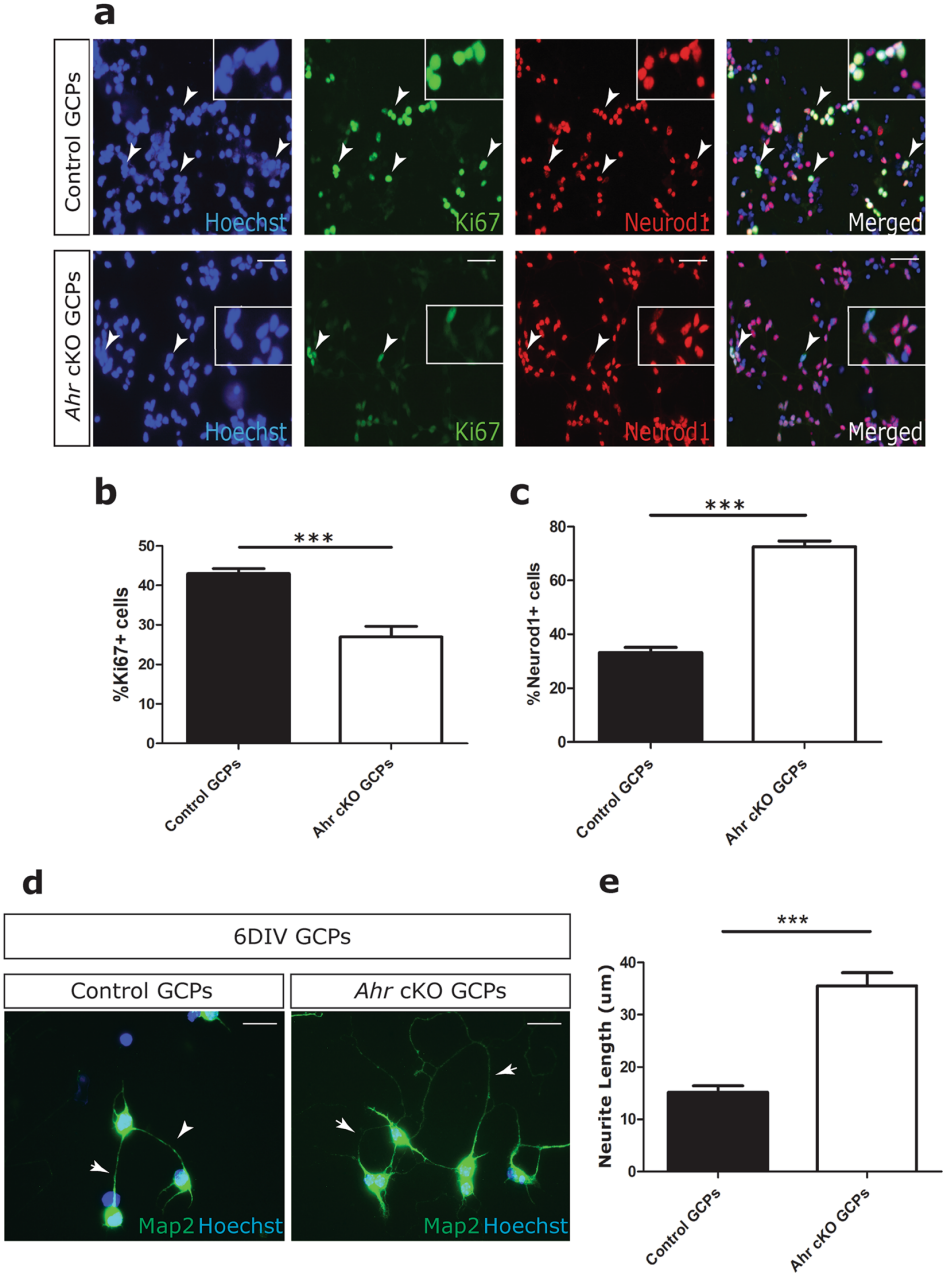
*Ahr* regulates the balance between proliferation and differentiation in GCPs. (**a**) Isolated P7 GCPs were cultured for 24 hours in the presence of exogenous SHH. Each cerebellum was processed independently (without pooling) from 3 control and 3 cKO littermates and the experiment repeated three times on separate occasions with independent litters. The data shown is the average of the three experiments with 9 WT and 9 cKO cerebella in total. Panels show immunostaining for Ki67 (green), Neurod1 (red) and Hoechst counterstained nuclei (blue). Merged panels show composite images. White arrows indicate Ki67+ cells. (**b**) Quantification of %Ki67 positive cells from a. (**c**) Quantification of %Neurod1 positive cells from a. (**d**) Isolated P7 GCPs were seeded in 6 well plates and allowed to differentiate in the absence of SHH, before immunocytochemical staining was performed for Map2 (green), followed by neurite tracing and quantification after 6 days *in vitro* (6 DIV). White arrows indicate neurites extending from granule neuron soma. (**e**) Quantification of neurite length (um). Data in panels b, c and e were analyzed by Student’s t test (p < 0.001 (***), p < 0.01 (**), p < 0.05 (*)). Bars represent mean values +/− SEM. Scale bars: 10 um (**a**,**d**).

We confirmed that this proliferative deficit was retained *in vitro*. *Ahr*-deficient, primary GCPs isolated from P7 *Ahr* cKO mice proliferated less compared to control GCPs (Fig. 1a,b). Furthermore, we found that more *Ahr*-deficient GCPs commenced differentiation as evidenced by expression of the definitive differentiation marker Neurod1 after a 24 hour culture period, compared to controls (Fig. 1a,c). A substantial fraction of GCPs displayed positivity for both Ki67 and Neurod1 (Fig. 1a), which can be expected to occur at 24 hours as cells are transitioning from a proliferative to a terminally differentiated state. To determine whether *Ahr*-deficient cells matured faster, primary GCPs were cultured in the absence of exogenous Sonic hedgehog (SHH) for six days *in vitro* and neurite length measured as an indicator of granule cell differentiation^27^. Map2 was used as a marker for neurites due to its importance in stabilizing microtubule activity in mature neurons^28^. *Ahr*-deficient GCPs displayed >2-fold increase in neurite length on average compared to controls (Fig. 1d,e), confirming a role for *Ahr* in suppressing GCP differentiation and maturation.

**Figure 2:**
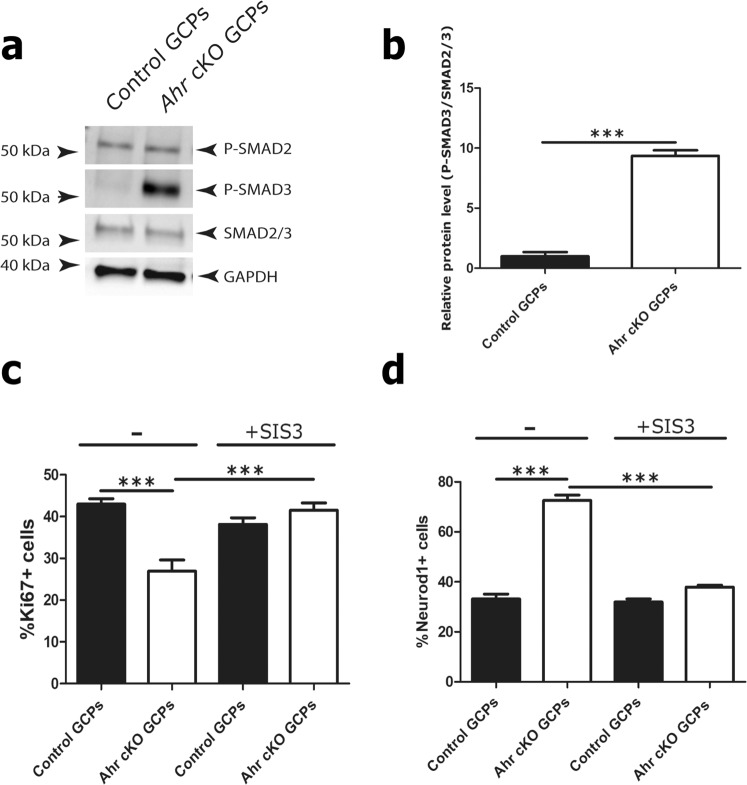
Loss of *Ahr* promotes GCP cycle arrest through enhanced activation of the TGF-β/SMAD3 axis. (**a**) Western blots of total cell lysates of isolated P7 GCPs from control and *Ahr* cKO cerebella with antibodies specific to phosphorylated S423/S425 residues of SMAD3 and phosphorylated S465/467 residues of SMAD2 proteins as well as antibodies against total SMAD2/3 and GAPDH (loading controls). Molecular weight markers are indicated on the left. (**b**) Quantification of band optic density for phosphorylated SMAD3, relative to total SMAD2/3, normalized to GAPDH levels. (**c**) Quantification of %Ki67 positive cells in non-treated (−) and 1 uM SIS3 (+SIS3) treated cultures. (**d**) Quantification of %Neurod1 positive cells. Data shown in (**c**,**d**) is representative of GCPs (on average 100 cells counted from 4 different fields of view from triplicate wells) isolated from 3 animals of each genotype, cultured for 24 hours in triplicate. Data was analyzed by Student’s t test (p < 0.001 (***), p < 0.01 (**), p < 0.05 (*)). Bars represent mean values +/− SEM.

To identify molecular pathways that were altered in *Ahr*-deficient cells, we performed immunoblots of lysates from purified GCPs to detect activated signalling mediators. For instance, as AHR has been shown to regulate the TGFβ-SMAD signalling pathway in several other contexts^29^, including brain tumours^30^, we assayed for the activated, phosphorylated (S423 and S425) form of SMAD3, an intracellular mediator of TGFβ receptor activation^31^. P-SMAD3 levels were elevated several-fold in *Ahr* cKO GCPs compared to control GCPs (Fig. 2a,b). In comparison, neither P-SMAD2 levels, nor the total amount of SMAD2 and SMAD3 proteins were altered in these cells (Fig. 2a). To determine whether SMAD3 hyperactivation was responsible for the altered phenotype of *Ahr*-deficient GCPs, primary GCPs from control and *Ahr* conditional mutant mice were cultured *in vitro* in the presence of exogenous SHH, with or without the selective SMAD3 inhibitor SIS3^32^. After 24 hours in culture, the fraction of proliferating and differentiating cells was quantified after immunostaining with antibodies to Ki67 and Neurod1, respectively. SIS3 treatment had no effect on the proliferation or differentiation of control GCPs in culture, suggesting that TGFβ-SMAD3 signalling is not an essential regulator of the proliferation or differentiation of these cells. However, the proliferative deficit of *Ahr*-deficient cells was fully rescued by SIS3 treatment, as was the tendency of these cells to differentiate (Fig. 2c,d). SIS3 treatment did not impact total GCP cell numbers over the course of 24 hours (Fig. S4a).

Together, these findings identify a role for AHR in keeping SMAD3 activation in check during normal GCP development. We conclude that SMAD3 hyperactivation is at least in part responsible for the proliferative deficit and enhanced differentiation of *Ahr*-deficient GCPs.

### AHR supresses tumour progression in a SHH medulloblastoma mouse model

**Figure 3:**
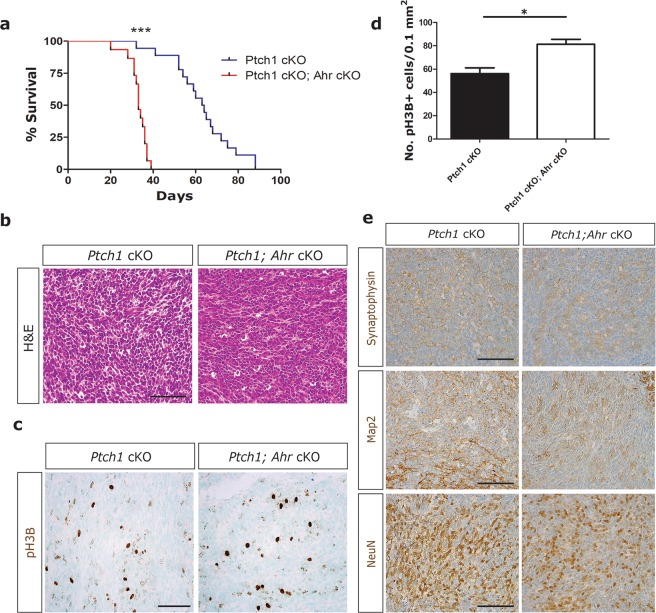
*Ahr* deletion in GCPs exacerbates tumorigenesis in a mouse model of SHH medulloblastoma. (**a**) Kaplan-Meier survival curves for *Math1cre; Ptch1*^*f/f*^ (*Ptch1* cKO; n = 19) and *Math1cre; Ahr*^*f/f*^*; Ptch1*^*f/f*^ (*Ptch1* cKO; *Ahr* cKO; n = 15) mice. (**b**) Representative haematoxylin/eosin (H&E) staining showing classic histology in both *Ptch1* cKO and *Ptch1* cKO; *Ahr* cKO medulloblastomas. (**c**) Immunohistochemical staining for mitotic marker pH3B (phosphorylated histone H3B) in brown. (**d**) Quantification of pH3B+ cells/mm^2^ of tumour (4 non-adjacent sections from 3 tumours of each genotype). (**e**) Immunostaining for neural differentiation markers synaptophysin, Map2 and NeuN (brown). Statistical comparison of survival curves in panel (a) was performed by log-rank test (p < 0.001 (***)). Data shown is of a single tumor assessed from each animal (n = 3 WT and 3 cKOs), as each animal presented with a single large tumour. Proliferation data in panel (d) was analyzed by Student’s t test (p < 0.05 (*)). Bars represent mean values +/− SEM. Scale bars: 50 um.

Cerebellar GCPs have been identified as the cell of origin for SHH medulloblastoma^33,34^. In mice, the conditional deletion of *Ptch1*, encoding the SHH receptor Patched1, which functions as an inhibitor of the SHH pathway, results in hyper-proliferation of GCPs, their rapid transformation and lethal medulloblastoma development in 100% of animals^35^. To determine whether *Ahr* has a role in SHH medulloblastoma, we deleted *Ahr* in these cells together with *Ptch1*. As previously reported^35^, 100% of *Math1cre; Ptch1*^*f/f*^ (*Ptch1* cKO) animals succumbed to medulloblastoma within 3 months of age, with a median survival of 63.5 days (Fig. 3a). By comparison, all *Math1cre; Ptch1*^*f/f*^*; Ahr*^*f/f*^ (*Ptch1* cKO Ahr cKO) animals died within 40 days of age, with a median survival of 33 days (Fig. 3a). The shorter survival time of animals with *Ahr*-deficient GCPs was highly significant (p < 0.0001, log-rank test). All animals presented with large medulloblastoma tumours with characteristic classic histology (Fig. 3b).

### *Ahr*-deficient medulloblastoma tumours have an undifferentiated phenotype

To characterize the salient features that distinguish *Ahr*-deficient from control tumours, we compared cell proliferation and differentiation. Overall, cellular proliferation was slightly, but significantly increased in *Ahr*-deficient tumours compared to control tumours (Fig. 3c,d). A comparison of control and *Ahr*-deficient tumours for steady-state levels of neuronal differentiation markers did not reveal any obvious difference in the levels of differentiation in *Ahr*-deficient tumours (Fig. 3e).

### *Ahr* controls cancer-propagating cell proliferation and differentiation via TGFβ-SMAD3 inhibition

**Figure 4:**
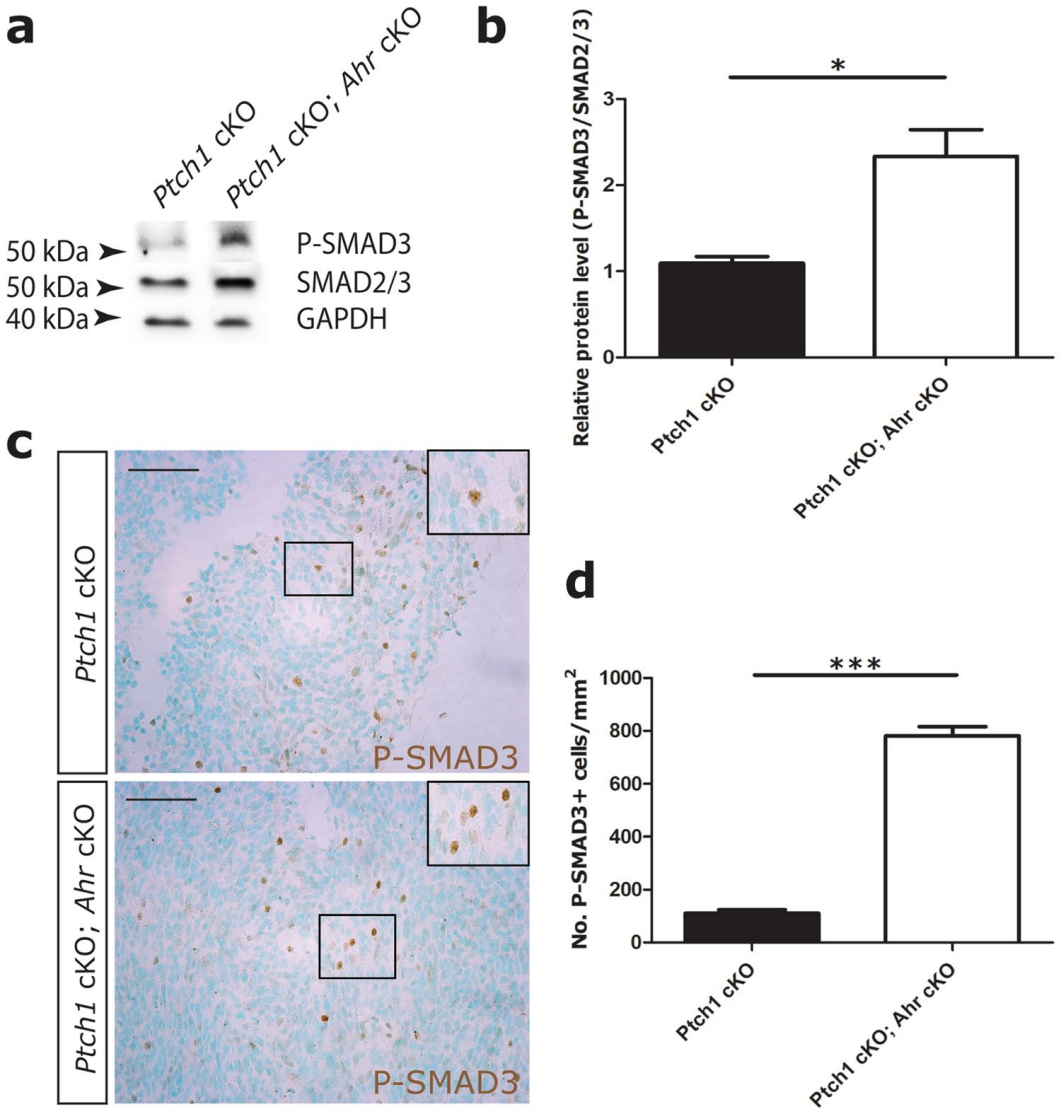
SMAD3 phosphorylation is increased in *Ahr*-deficient SHH medulloblastomas. (**a**) Western blots of total cell lysates of isolated end stage medulloblastoma tissue with antibodies specific to phosphorylated S423/S425 residues of SMAD3 (P-SMAD3), total SMAD2/3 and GAPDH proteins. (**b**) Quantification of band optic density for phosphorylated SMAD3 relative to SMAD2/3 levels, normalized to GAPDH levels. Data is representative of 3 animals/genotype. (**c**) Immunohistochemical staining of end stage tumour sections with P-SMAD3 antibody (in brown) with methyl green counterstaining nuclei. (**d**) Quantification of P-SMAD3+ cells/mm^2^ of tumour tissue (4 non-adjacent sections from 3 tumours of each genotype). Data was analyzed by Student’s t test (p < 0.001 (***)). Bars represent mean values +/− SEM. Scale bars: 100 um.

Next, we asked whether AHR also inhibited SMAD3 activation in SHH medulloblastoma. Immunoblot analysis of tumour lysates found elevated levels of P-SMAD3 in *Ahr*-deficient samples, compared to controls (Fig. 4a,b). Levels of total SMAD2/3 protein were also increased in *Ahr* cKO tumours (Fig. S3c,d). Immunostaining of medulloblastoma tissue revealed a salt and pepper distribution of P-SMAD3 positive cells in the tissue, suggesting that only a subset of cells in *Ahr* cKO tumours responded to TGFβ signals at a given time point (Fig. 4c). When comparing P-SMAD3 immunostaining between control and *Ahr* cKO medulloblastomas, we found that the number of cells with detectable P-SMAD3, as well as the intensity of P-SMAD3 staining in these cells, were increased in *Ahr*-deficient tumour tissue (Fig. 4d).

**Figure 5:**
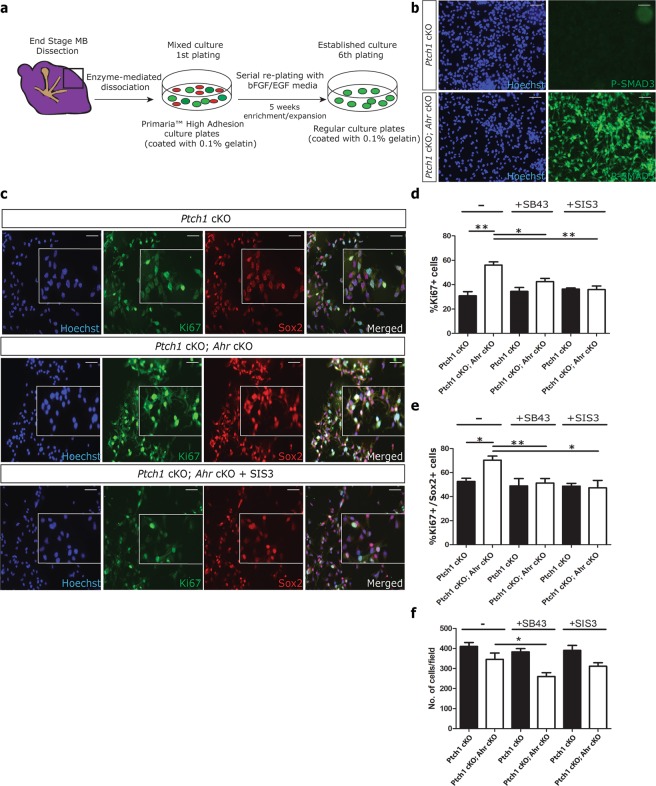
*Ahr* suppresses SHH MB CPC proliferation by repressing SMAD3 activation. (**a**) Procedure involved in isolation and maintenance of MB CPCs^38^. (**b**) Immunocytochemical staining of proliferating MB CPC cultures for phosphorylated SMAD3 (green) with Hoechst counterstained nuclei (blue). (**c**) The same cultures were treated for 24 hours with or without TGFβ pathway inhibitors and stained for Ki67 (green), Sox2 (red) and Hoechst (blue). (**d**) Quantification of %Ki67+ cells in non-treated (−), 500 nM SB43 (+SB43) and 1uM SIS3 (+SIS3) treated cells. (**e**) Quantification of %Ki67+; Sox2+ (double positive) cells in each culture condition. (**f**) Quantification of total cell number/field of view based on Hoechst staining. On average 100 cells were counted from 4 different fields of view from triplicate wells of each condition. Data shown is representative of CPCs isolated from 2 animals/genotype with experiments performed in triplicate for each. Data was analyzed by Student’s t test (p < 0.01 (**), p < 0.05 (*)). Bars represent mean values +/− SEM. Scale bars: 10 um (**b**,**c**).

Given previous studies linking *Ahr* function with maintenance of the cancer-propagating cell (CPC) compartment^36,37^ and the evidence supporting a role for Sox2+ CPCs in promoting SHH medulloblastoma aggressiveness^20,21^, we decided to investigate whether the elevated SMAD3 activity in *Ahr*-deficient medulloblastoma modulated important CPC properties. To achieve this, we established primary cultures of CPCs from end stage medulloblastomas and maintained these cells in serum-free stem cell medium supplemented with growth factors, as described previously^38^ (Fig. 5a). As expected, the majority (70–80%) of medulloblastoma CPCs were positive for the neural stem cell marker Sox2 and TGFβ inhibitor treatments had no effect on the proportion of Sox2 expressing cells (Fig. S2a), indicating that Sox2+ cell identity or Sox2 expression were not dependent on TGFβ signalling. Immunostaining of these cells revealed that all *Ahr*-deficient medulloblastoma CPCs were strongly positive for p-SMAD3, compared to control culture that did not display SMAD3 activation (Fig. 5b). This finding suggested that the AHR pathway suppressed TGFβ-SMAD3 signalling in Sox2+ cells.

In agreement with observations in tumour sections (see Fig. 3d), *Ahr*-deficient cultures contained significantly higher numbers of proliferating cells compared to controls (Fig. 5c,d). Inclusion of the SMAD3 phosphorylation inhibitor SIS3^32^ in these proliferating cultures reduced levels of cycling to near control levels (Fig. 5c,d). Quantification of the proportion of cycling (Ki67+) Sox2+ CPCs revealed the same trend (Fig. 5e). These data implicating TFGβ/SMAD3 signalling in medulloblastoma CPC proliferation were corroborated by treating the cells with the selective TGF-β receptor I inhibitor SB-431542 (SB43)^39^ (Fig. 5d,e). Treatment of *Ahr*-deficient CPCs with both SIS3 and SB43 inhibitors led to a significant reduction in cell numbers (~25% with SIS3 and ~35% with SB43), suggesting that TFGβ/SMAD3 signalling may function by promoting CPC survival. Together, these findings implicated the TGF-β/SMAD3 pathway in mediating the effect of AHR signalling on proliferation and cell survival of medulloblastoma CPCs.

**Figure 6:**
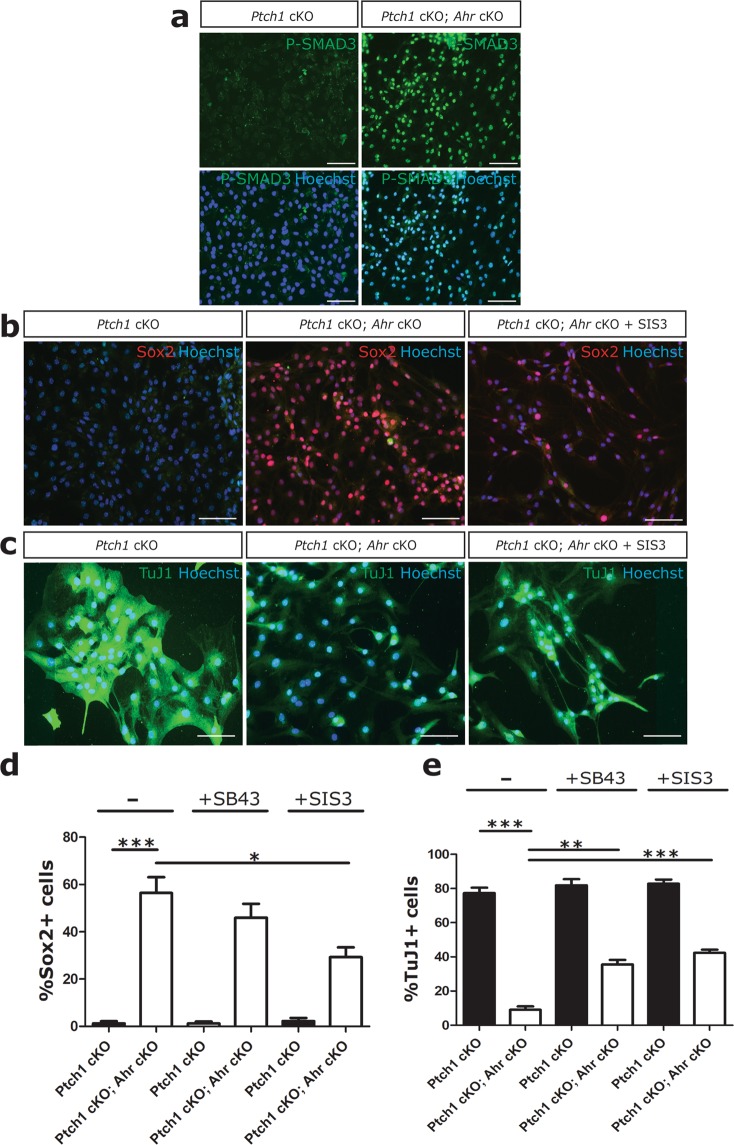
*Ahr*-deficient MB CPCs resist differentiation partly through SMAD3 activation. (**a**) Immunocytochemical staining of 7 DIV (day *in vitro*) differentiation cultures for P-SMAD3 (green) and Hoechst (blue). Note increased P-SMAD3 signal intensity in *Ahr* cKO cultures. (**b**) Staining of *Ptch1* cKO and *Ptch1* cKO; *Ahr* cKO cultures with/without 1uM SIS3 for Sox2 (red). (**c**) Staining of the same cultures for TuJ1/βIII tubulin (green). (**d**) Quantification of %Sox2+ cells in all culture conditions. (**e**) Quantification of %TuJ1+ cells in all culture conditions. On average 100 cells were counted from 4 different fields of view from triplicate wells of each condition. Note increased number of Sox2+ and reduced number of TuJ1+ cells in *Ahr* cKO CPCs and partial rescue with SIS3 treatment. Data shown is representative of CPCs isolated from 2 animals/genotype performed in triplicates. Data was statistically analyzed by Student’s t test (p < 0.001 (***), p < 0.01 (**), p < 0.05 (*)). Bars represent mean values +/− SEM. Scale bars: 10 um (**a**–**c**).

To ask whether *Ahr* deletion prevented medulloblastoma CPC differentiation via TGFβ-SMAD3 signalling, cells were transferred to a culture medium that promotes the differentiation of these cells^38^. Under these differentiation conditions, control cells completely lost expression of Sox2 with 7 days, while approximately 60% of *Ahr*-deficient medulloblastoma CPCs retained high levels of Sox2 expression, indicative of a retention of an undifferentiated phenotype (Fig. 6b,d). Control *Ptch1* cKO CSC cultures maintained under these differentiation conditions were still characterised by low levels of SMAD3 activity, compared to *Ptch1* cKO; *Ahr* cKO cells that had high levels of nuclear P-SMAD3 (Fig. 6a).

Treatment of *Ahr* cKO cultures with either TGFβ/SMAD inhibitors (SB43 and SIS3) over 7 days significantly reduced the proportion of Sox2+ cells by 30–40% (Fig. 6d). Inhibitor treatments had no effect on Sox2+ cells in control cultures. To confirm and compare levels of differentiation, both control and *Ahr* cKO CPC cultures were stained for TuJ1/βIII tubulin, a marker of immature neuron differentiation^38^. After 7 days of differentiation, nearly 80% of control *Ptch1* cKO CPCs were positive for TuJ1, while only 5–10% of *Ptch1* cKO; *Ahr* cKO CPCs were positive for this marker (Fig. 6c,e). TGFβ inhibition increased TuJ1 expression in *Ahr* cKO cultures, while having no effect on control CPC differentiation (Fig. 6c,e). The rescue effect on *Ahr* cKO cultures was partial, suggesting involvement of other pathways beside TGFβ/SMAD3 in promoting resistance to differentiation in *Ahr* cKO CPCs.

Taken together, these studies suggested that *Ahr* deletion in SHH medulloblastoma promoted CPC fate via induction of TGFβ/SMAD3 signalling. Correspondingly, TGFβ/SMAD3 inhibition promoted CPC differentiation.

### SHH medulloblastoma patients with high levels of *AHRR* expression show reduced survival

**Figure 7:**
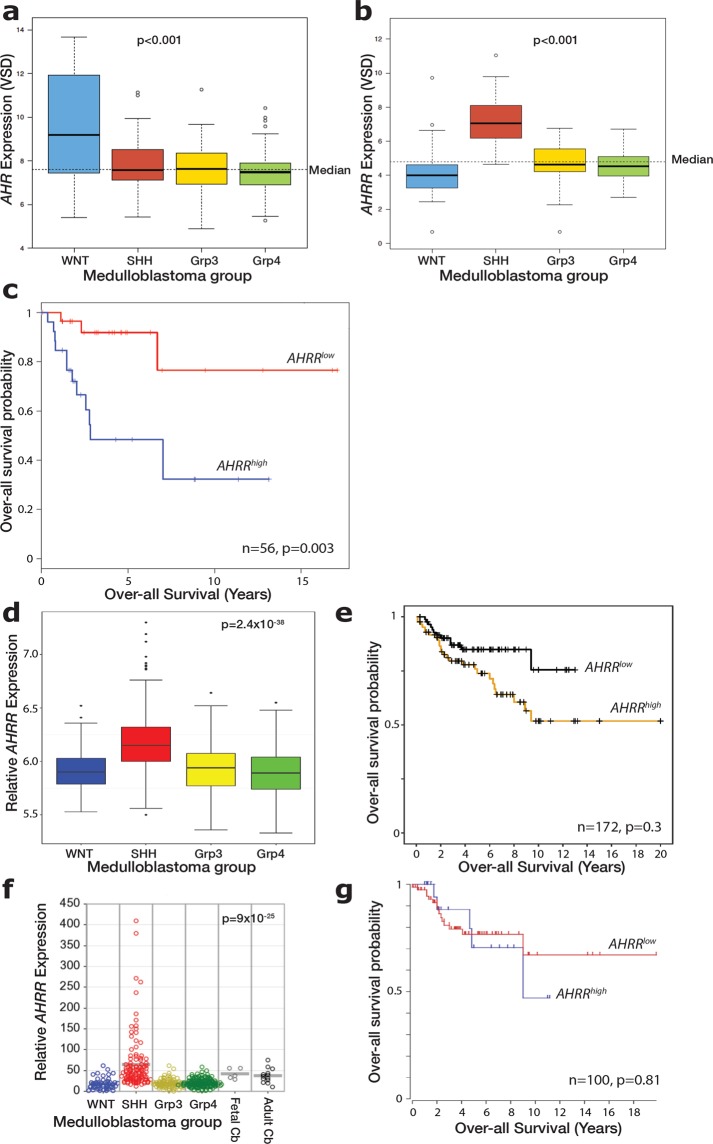
Significantly reduced survival of patients with SHH MB expressing high levels of AHR repressor (*AHRR*). (**a**) *AHR* gene expression comparison across different medulloblastoma subgroups (WNT (n = 28), SHH (n = 58), Grp 3 (n = 59), Grp 4 (n = 95)) from the Clifford cohort. Note significant difference in WNT subgroup. (**b**) *AHRR* gene expression comparison across different medulloblastoma subgroups. Note significant difference in SHH subgroup. (**c**) Kaplan-Meier survival curves from Clifford group patient dataset showing survival rate comparison between SHH MB patients with high and low expression of *AHRR* for the 56 SHH patients for whom follow-up data was available. (**d**) *AHRR* gene expression comparison across different medulloblastoma subgroups (WNT (n = 70), SHH (n = 223), Grp 3 (n = 144), Grp 4 (n = 326)) from the SickKids cohort^6^. Note significant difference in SHH subgroup. (**e**) Kaplan-Meier survival curves from Taylor group patient dataset showing survival rate comparison between SHH MB patients with *AHRR* expression below or above median levels. Note similar trend with Clifford dataset of reduced survival probability with heightened *AHRR* expression. (**f**) *AHRR* gene expression comparison across different medulloblastoma subgroups (WNT (n = 53), SHH (n = 112), Grp 3 (n = 94), Grp 4 (n = 164)) from the Kool cohort. Note significant difference in SHH subgroup. (**g**) Kaplan-Meier survival curves from Kool group patient dataset showing survival rate comparison between SHH MB patients with *AHRR* expression below or above median levels. Data in a,b and d were statistically analyzed by an Anova test and data in c,e and g by log rank test.

To determine if our findings of a tumour-suppressive role for the AHR pathway may have direct clinical relevance, we examined the expression of *AHR* in a cohort of human medulloblastomas, profiled by the Clifford group, with the following subgroup distribution: WNT (n = 28), SHH (n = 58), Grp 3 (n = 59), Grp 4 (n = 95). *AHR* gene expression was significantly higher in the WNT subgroup, with no difference between other subgroups (Fig. 7a). Furthermore, *AHR* expression levels did not correlate with patient survival in any subgroup (data not shown). Intriguingly, we found that the expression of the *AHRR* gene, which encodes an AHR Repressor protein, was elevated specifically in the SHH subgroup (Fig. 7b). When examining the association between AHRR expression in SHH tumours and patient survival, we found a statistically significant reduction in patient survival in medulloblastomas with high (>median) *AHRR* expression (Fig. 7c). This relationship between *AHRR* expression and survival was specific to the SHH subgroup with no associations found in other subgroups (data not shown). These findings are consistent with a model whereby a reduction of the AHR pathway, either via reducing Ahr expression (as in our mouse model), or increased *AHRR* expression in human tumours, is associated with more aggressive SHH medulloblastoma tumours and reduced patient survival.

Finally, we asked if we could replicate our human medulloblastoma findings in an independent, larger patient cohort. An analysis of 172 SHH tumours from the Taylor group in Toronto, confirmed both the increased expression of *AHRR* in SHH tumours, compared to other subgroups (Fig. 7d), as well as reduced survival of patients with high *AHRR* expression (Fig. 7e). The same *AHRR* expression profile was observed across medulloblastoma subgroups in a third, independent cohort profiled by the Kool group in Heidelberg (Fig. 7f), however no correlation between *AHRR* expression levels and survival was observed in this particular cohort of SHH medulloblastomas (Fig. 7g).

## Discussion

Here we identified a critical role for *Ahr* in preventing activation of the TGFβ mediator *SMAD*3, both in primary cerebellar GCPs and GCP-derived SHH meduloblastoma. We further found that CPCs derived from *Ahr*-deficient tumours exhibited very high levels of P-*SMAD*3 compared to tumours with intact *Ahr*. *Ahr* deletion in GCPs together with the cancer-initiating *Ptch1* gene deletions dramatically reduced survival, identifying a potent tumour-suppressive role for *Ahr*. CPCs from these *Ahr*-deficient tumours were refractory to differentiation *in vitro*. Most importantly, pharmacological inhibition of the TGFβ-*SMAD*3 pathway was sufficient to drive *Ahr*-deficient CPCs towards differentiation, identifying this pathway as a potentially viable therapeutic target for aggressive medulloblastoma subtypes with reduced AHR pathway activity and elevated TGFβ-*SMAD*3 signalling. Transcriptomic analyses of human SHH medulloblastomas indeed identified a substantial subset of SHH primary tumours with high *AHRR* expression and poor prognosis in two independent patient cohorts. As these aggressive SHH medulloblastoma subtypes are highly resistant to conventional therapies, future studies to fully characterise these *AHRR*^*high*^ tumours, establish to what extent they resemble *Ahr*-deficient SHH tumours in the mouse, and explore the potential of TGFβ-SMAD3 pathway inhibition will be important.

Several mechanistic questions remain to be answered. Exactly how *Ahr* deletion leads to specific activation of SMAD3, and not SMAD2 is not known. This apparently exquisite specificity argues against a general induction of TGFβ ligands or membrane receptors, which would be expected to activate both SMAD2 and SMAD3. The increase in total SMAD2/3 protein levels suggests Ahr might also function by either suppressing SMAD2/3 transcription in the tumour context or promoting SMAD2/3 proteolytic turnover.

Our findings further support the idea that the role of the AHR pathway is highly context-specific. In primary GCPs, *Ahr* deletion leads to reduced proliferation and enhanced differentiation, while in SHH medulloblastomas derived from these cells, *Ahr* deletion has the opposite effect. These observations are particularly important in the light of a previous study showing that *Ahr* knockdown in the SHH-like medulloblastoma cell line DAOY resulted in reduced cell proliferation^25^.

It should be noted that other genes in the AHR pathway have been implicated in medulloblastoma. In particular, the *Arnt* (Aryl hydrocarbon receptor nuclear translocator) gene, which encodes an AHR interacting protein necessary for its function, can promote leptomeningeal metastatic dissemination when overexpressed in SHH medulloblastomas in the mouse^40^. Whether these effects are as a result of modulation of the AHR pathway remains to be determined.

It is intriguing to consider our observations in SHH medulloblastoma CPCs in the context of other brain tumours. Gramatzki *et al*. have shown that AHR inhibition in glioma cells also resulted in upregulation of the TGFβ-SMAD pathway^30^. Elevated TFGβ-SMAD signalling promotes glioma stem cell self-renewal and is associated with more aggressive gliomas and poorer survival^41,42^. Together, these findings imply some conservation in the role of AHR in repressing the TGFβ-SMAD pathway in brain tumour stem cells and suggest the possibility that inactivation or repression of this pathway may represent a mechanism whereby tumour cells retain a stem-like character and become resistant to differentiation. This study did not assess the effects of TGFβ-SMAD pathway modulation on apoptosis, however a significant decrease in *Ahr* cKO CPC number observed with SB43 and SIS3 treatment suggests that enhanced CPC survival may be an important mechanism of increased tumorigenicity in *Ahr*-deficient SHH medulloblastoma.

The role of the TGFβ-SMAD pathway specifically in medulloblastoma remains unclear. The present study links hyper-activation of this pathway in SHH medulloblastoma with resistance to differentiation and poor prognosis. This finding is in disagreement with Aref *et al*. who suggested that nuclear SMAD3 localisation as a proxy read-out of SMAD3 activation in SHH medulloblastoma samples correlated with good prognosis^43^. This study only assessed SHH medulloblastomas from 35 patients and using nuclear localisation of SMAD3 as a read-out of pathway activation may not be ideal. Clearly, a larger study to assess P-SMAD3 immunoreactivity will be important. Interestingly, elevated TGFβ signalling has also been implicated as a driver of Group 3 medulloblastoma^44^, suggesting that this pathway may also play important roles in other medulloblastoma subtypes.

Our human transcriptomic analyses identified *AHRR* expression as a novel biomarker for aggressive SHH medulloblastoma in two of three independent cohorts investigated. Demographic differences and the mixed therapies deployed within these retrospective cohorts may explain the lack of consistent observations in all three cohorts, and these encouraging initial findings now require prospective validation in clinical trials-based cohorts. Determining to what extent high AHRR expression in human SHH medulloblastoma is associated with elevated TGFβ-SMAD3 signalling will be an important next step to identify patients that may benefit from TGFβ-SMAD3 inhibition and/or AHR agonist therapies.

## Materials and Methods

### Animals

*Math1cre*^45^, *Ptch1 flox*^46^, and *Ahr flox*^47^ mouse lines have been described and were genotyped by PCR using tail or ear DNA extracted using proteinase K digestion or the HotSHOT method^48^. PCR primers used are indicated in the table below. *Ptch1* cKO mice were generated by crossing *Math1cre* and *Ptch1*^*f/f*^ mice, followed by crosses of the resultant *Math1cre; Ptch1*^*f/+*^ with *Ptch1*^*f/f*^ animals. *Ahr* cKO medulloblastoma mice were generated by crossing *Math1cre* and *Ahr*^*f/f*^*; Ptch1*^*f/f*^ mice followed by crosses of the resulting *Math1cre; Ahr*^*f/+*^; *Ptch1*^*f/+*^ mice with *Ahr*^*f/f*^; *Ptch1*^*f/f*^ animals. Mice were bred and maintained according to Home Office regulations in New Hunt’s House, Biological Services Unit, King’s College, London. For postnatal stages the day of birth was designated postnatal day 0 (P0). All mice on tumour watch were monitored closely for symptoms of medulloblastoma and were sacrificed upon discovery of symptoms such as hydrocephalus, weight loss and ataxic gait^49^. The institutional Local Ethical Review Panel and the UK Home Office approved all experimental procedures (Project licence numbers: 70/7506 and P8DC5B496). All procedures were carried out by a personal licence holder.

**Table:** 

Gene	Forward Primer	Reverse Primer
*Cre*	5′-CCTGGAAAATGCTTCTGTCCG-3′	5′-CAGGGTGTTATAAGCAATCCC-3′
*Ahr*^*flox*^	5′-CAGTGGGAATAAGGCAAGAGTGA-3′	5′-GGTACAAGTGCACATGCCTGC-3′
*Ptch1*^*flox*^	5′-CCACCAGTGATTTCTGCTCA-3′	5′-AGTACGAGGCATGCAAGACC-3′

Thermal cycles for *Cre* and *Ptch1*^*flox*^ genotyping were as follows: 94 °C, 10 minutes; 40× (94 °C, 45 sec; 57 °C, 45 sec; 72 °C, 60 sec); 72 °C, 7 minutes. Thermal cycles for *Ahr*^*flox*^ genotyping were: 94 °C, 3 minutes; 35 × (94 °C, 30 sec; 69 °C, 60 sec; 72 °C, 60 sec); 72 °C, 2 minutes.

### Tissue processing and histology

Brains were dissected in ice cold PBS and fixed in 4% paraformaldehyde (PFA) at 4 °C. Samples were dehydrated, cleared and infiltrated with paraffin wax using a Leica ASP300 Tissue Processor, followed by sagittal embedding in paraffin. Paraffin tissue blocks were sectioned using a microtome (Leica RM2145). Serial sections were cut at 4–10 μm thickness and mounted onto glass slides (SuperfrostPlus®, VWR^TM^).

### Immunohistochemistry

#### 3, 3′-diaminobenzidine (DAB) immunohistochemistry

Sections were deparaffinised and rehydrated through a series of graded ethanols to PBS. Endogenous peroxidases were blocked in a solution containing 3% H_2_O_2_ (stock 30%) and 10% methanol in PBS for 15 minutes and were subsequently rinsed with dH2O. Sections were then heated in the microwave at full power in a 10 mM sodium citrate solution (pH 6.0) (4 × 5 mins) to break methylene bridges associated with the fixation process and expose antigenic sites. After cooling for 20 minutes at room temperature, cells were then permeabilised using 0.2% PBSTx (Triton®X-100 in PBS) (1 × 10 minutes) and non-specific antibody binding was blocked by incubating slides in 10% goat serum in PBSTx for 1 hour at room temperature. Sections were then incubated with the primary antibody diluted in 5% goat serum in PBSTx overnight at 4 ^o^C. The following day unbound antibody was removed using three 10 minute 0.1% PBSTx washes and sections were then incubated in the appropriate biotinylated secondary antibody (1/200) diluted in 5% goat serum in 0.1% PBSTx. After 1 hour at room temperature unbound secondary antibody was washed off using PBS (3 × 10 minutes). The signal was then amplified using the VECTASTAIN Avidin Biotin Complex (ABC) kit by incubating sections in a solution containing 1/200 dilutions of A and B in PBS for 1 hour at room temperature. Sections were then washed in PBS (3 × 10 minutes) and visualised with 0.03% 3, 3′- diaminobenzidine (DAB) substrate (stock 30% in Tris-HCl)/ 0.0003% hydrogen peroxide (stock 30%). After visualization sections were then counterstained with Methyl Green (1 × 5 minutes), briefly washed under running water and then dehydrated in a series of ascending ethanols, cleared in xylene, mounted using DPX and imaged with a Nikon Eclipse 80i microscope.

### Immunofluorescence

#### Paraffin sections

Sections were deparaffinised and rehydrated as above and washed with PBS (2 × 5 mins). Antigen retrieval was performed by heating sections in a solution of 10 mM sodium citrate (pH 6.0) for 4 × 5 minutes at full power in the microwave. Sections were then left to cool to room temperature for 20 minutes. Tissue was permeabilized in 0.2% PBSTx and blocked in 10% heat inactivated goat serum in PBSTx for 1 hour before incubating in primary antibody in 5% goat serum in PBSTx overnight at 4 ^o^C. The following day, unbound antibody was removed using 3 × 10 minute PBS washes. Slides were incubated with Alexa-Fluor-labelled secondary antibodies (1/200, Life technologies) in 5% goat serum in PBSTx. Unbound secondary antibody was then washed off with 3 × 10 minute PBS washes. The nuclear counterstain, 4′-6-diamidino-2-phenylindole (Dapi) (1:5000, Invitrogen) was added to the final wash and slides were mounted using Citifluor (www.citifluor.com). Tumour histology for differentiation markers Synaptophysin, MAP2 and NeuN were performed on a Ventana Medical System Benchmark automated immunostainer as described^50^.

#### Fixed cells

Cells fixed on coverslips with 4% PFA were initially washed with 2 × 10 minute PBS washes. They were then permeabilized in 0.2% PBSTx and blocked in 10% heat inactivated goat serum in PBSTx for 1 hour before incubating in primary antibody in 5% goat serum in PBSTx overnight at 4oC. The following day, unbound antibody was removed using 3 × 10 minute PBS washes. Slides were incubated with Alexa-Fluor-labelled secondary antibodies (1/200, Life technologies) in 5% goat serum in PBSTx. Unbound secondary antibody was then washed off with 3 × 10 minute PBS washes. The nuclear counterstain, 4′-6-diamidino-2-phenylindole (Dapi) (1:5000, Invitrogen) was added to the final wash and slides were mounted using Citifluor (www.citifluor.com). Fluorescent images were captured using Nikon Eclipse 80i with Nikon Y-QT Hamamatsu C4742-95 camera.

### Antibodies

#### Primary

Primary antibodies used were as follows: rabbit anti-GAPDH (1/2500, Abcam, ab9485), rabbit anti-phospho histone H3B (1/200, NEB, 9701S), rat anti-BrdU (1/50, Abcam, ab6326), mouse anti-BrdU (1/100, BD, 347580), mouse anti-NeuN (1/2000, Chemicon), mouse anti-Map2 (1/500, Chemicon), rabbit anti-Ki67 (1/1000, Abcam, ab15580), rabbit anti-P-SMAD2/3 (1/1000, NEB, D27F4), rabbit anti-SMAD2/3 (1/1000, NEB, 3102S), mouse anti-Neurod1 (1/100, Abcam, ab60704), rabbit anti-synaptophysin (Pe-dilued, Zymed, 080130), rabbit anti-P-SMAD3 (1/100, Abcam, ab52903), rabbit anti-TuJ1 (1/500, Abcam, ab18207) and mouse anti-Sox2 (1/500, Abcam, ab79351).

#### Secondary

For western blots primary antibodies were detected using polyclonal goat anti-rabbit IgG horse radish peroxidase (HRP) conjugated secondary antibody (1/2000, Thermofisher, 65-6120). For DAB immunohistochemistry primary antibodies were detected using polyclonal goat anti-mouse IgG biotinylated secondary antibody (1/200, Dako, E0433), polyclonal goat anti- rabbit IgG biotinylated secondary antibody (1/200, Dako, E0432). For immunofluorescence primary antibodies were detected using Alexafluor-488 goat anti-rabbit IgG (Invitrogen, 1/200, A11034), Alexafluor-568 goat anti-mouse IgG (Invitrogen, 1/200, A21124), Alexafluor-488 goat anti-mouse IgG (Invitrogen, 1/200, A11001), Alexafluor-568 goat anti-rabbit IgG (Invitrogen, 1/200, A11011) secondary antibodies.

#### Western blots

Purified GCPs were isolated from P7 cerebella and either whole cell protein or subcellular fractions were prepared by lysing in N-PER lysis buffer (Thermofisher) or subcellular fractionation buffers (Thermofisher) respectively, following the manufacturer’s instructions. All buffers contained protease inhibitors (PMSF, Pepstatin A, Leupeptin, Aprotinin; Roche) and a phosphatase inhibitor cocktail (Sigma). Protein loading samples were made by diluting samples in Laemmli buffer containing 10% β-mercaptoethanol, followed by boiling at 95 °C for 5 minutes. Samples were loaded (10 µg total protein per lane) onto a Mini-PROTEAN pre-cast gel (Bio-Rad) and resolved using gel electrophoresis. Protein was transferred to a nitrocellulose membrane (Bio-Rad) which was then blocked in 5% non-fat milk powder (Bio-Rad) or 3% bovine serum albumin (BSA, Sigma) in TBS with 0.1% Tween-20 (TBST) for one hour at room temperature, followed by incubation with primary antibodies diluted in 3% BSA/TBST overnight at 4 °C. The next day the membranes were washed 3 × 10 mins in TBST, followed by incubation in secondary antibodies diluted in 5% non-fat milk powder in TBST for one hour at room temperature. Membranes were subsequently washed again 3 × 10 minutes in TBST and HRP was detected with Clarity ECL reagent (Bio-Rad) and the membranes imaged using a Bio-Rad ChemiDoc system. Relative protein quantity was calculated using Bio-Rad ImageLab software.

#### Cerebellar GCP isolation

GCPs were isolated as described previously^51^. The cerebella from P7 control and *Math1cre; Ahr*^*f/f*^ pups were dissected in ice-cold DPBS. The lobules that retain *Ahr* expression (IX + X) along with the flocculus and paraflocculus were removed. Each cerebellum was processed separately. Papain-I (100 U in 10 ml DPBS) was dissolved in 10 ml of DPBS at 37 °C. Once dissolved, 200 μl of DNaseI (12,500 U/ml, Sigma) was added and the Papain-I enzyme was activated by adding L-Cysteine (2 mg/10 ml). After adjusting the pH to using 2 N NaOH, cells were dissociated by incubating the cerebella in the Papain-I containing solution for 30 minutes at 37 °C followed by trituration in ovo solution (2 mg/ml ovomucoid, 125 U/ml DNaseI in DPBS). Dissociated cells were then centrifuged for 10 minutes at 1000 rpm. The supernatant was then aspirated and the pellet suspended in DPBS-BSA (DPBS containing 1% BSA). The suspension was then passed through a cell strainer before underlaying the solution containing the dissociated cells with 35% Percoll followed by 65% Percoll. Cells were then separated according to size by centrifugation (12 minutes, 2500 rpm). The layer constituting the interphase between the 35% and 65% Percoll layers, containing GCPs, was then removed and placed in a separate falcon tube containing 14 ml of DPBS-BSA. GCPs were counted using a haemocytometer, the solution containing GCPs was then centrifuged (10 minutes, 1400 rpm) and the supernatant aspirated.

#### Cell culture

Purified GCPs were cultured in Neurobasal media (Thermofisher) supplemented with B27 (Thermofisher), exogenous SHH, glutamine (Thermofisher) and penicillin/streptomycin (Sigma). The SHH came from supernatant obtained from conditioned media from HEK293T cells transfected with pcDNA3.1 ShhN plasmid. pcDNA3.1 ShhN was a kind gift from Philip Beachy (Addgene plasmid # 37680). Culture vessels were pre-coated with poly-D-lysine (Sigma) before cell seeding. Media was changed every other day to maintain growth conditions. Omission of exogenous SHH promoted differentiation. CPCs were isolated from end-stage tumours as described in Fig. 5a. CPCs were cultured as described in^38^. Briefly, enriched CPCs were cultured in Neurobasal media supplemented with B27 (Thermofisher), N2 (Thermofisher), bFGF (Peprotech), EGF (Peprotech), glutamine (Thermofisher) and penicillin/streptomycin (Sigma) to maintain growth conditions. Media was changed every other day in expanding cultures. Culture vessels were pre-coated with 0.1% gelatin (Sigma) before cell seeding. Upon switching the medium to 10% serum in DMEM (Thermofisher) the cells underwent differentiation. In experimental cultures SB-431542 (SB43) (Sigma) or SIS3 (Sigma) were added to some culture wells as part of a daily change of medium. SB43 functions as an inhibitor of the transforming growth factor-beta superfamily type I (TGFβRI) receptor^39^ while SIS3 is a specific inhibitor of SMAD3 phosphorylation and its interaction with SMAD4^32^.

#### Quantitative analysis

Ki67+ and Neurod1+ cells quantified in Figs. 1b,c and 2c,d were counted from four different fields of view/quadrants (top to bottom, left to right) from triplicate wells (of 24 well plates) of each condition. Hoechst counterstained cells were counted alongside and the final % positivity calculated and averaged for each condition. The Hoechst staining quantification is from the same fields with filter change. Neurites were traced using the Simple Neurite Tracer plugin in ImageJ and the path lengths of traced neurites from each cell in the field of view were converted to a micron scale before calculating the mean length. The starting point of the neurite was taken as the point of incidence from the cell soma. The data was assessed by a single, blinded researcher, and the experiment was performed in three independent instances from three separate control and cKO cerebella. Proliferation data in Fig. 3d was quantified by counting the number of pH3B+ cells in 100um x 100um squares from every 30th section (10um thick sections) of three tumours of each genotype (each from a separate animal), followed by averaging. The same method was used to obtain data in Fig. 4d. Ki67+, Sox2+ and TuJ1+ CPCs quantified in Figs. 5d,e, 6d,e were counted in the same manner as the GCP data described above. Fluorescent images were captured using Nikon Eclipse 80i with Nikon Y-QT Hamamatsu C4742-95 camera. Acquired fluorescent images from cell culture experiments were subjected to an identical intensity threshold, as judged by the investigator, in ImageJ (separately for each antigen) before counting positive cells. The experimenter was blinded for the IHC/ICC quantification in (Figs. 1, 3 and 4) and data quantification in the cell culture experiments (Figs. 2 and 5) was done and repeated by another researcher in double-blind fashion where neither of the researchers was aware of the culture conditions or group identity.

#### Image processing

Images were processed using Adobe Photoshop CC 2017 and figures assembled in Adobe Illustrator CC 2017.

### Statistics

Statistical analysis was carried out and graphs generated using GraphPad Prism 5®. Most data were analysed using a Student’s t-test with the exception of the Kaplan-Meier survival analysis which used the log-rank test or Cox regression analysis. P < 0.05 was considered significant.

#### Transcriptomic analyses from human data

Read counts for *AHR* and *AHRR* expression were produced by aligning paired end RNA-seq (~90 M read/sample Illumina HiSeq 2500) reads to HG19 genome using STAR-align^52^. Read counts were produced using HT-SEQ-count. DESeq 2 (R/Bioconductor) was used to normalise reads to library size and variance stabilised data (VSD) was generated using the vsd function. Statistical testing for differential expression across groups was performed using an ANOVA test. Affymetrix expression data were obtained from previously reported series through GEO accession numbers GSE10327^8^, GSE12992^53^, GSE37418^54^, GSE49243^55^ and published previously^56^. All data were MAS5.0 normalized and analysed using the genomics analysis and visualization platform R2 (http://r2.amc.nl). For survival analyses the Kaplan scanning tool in R2 was used that identifies the optimal cut off in expression in a dataset that results in the lowest log rank p value in overall survival analyses between the subset with high expression and the subset with low expression of the gene of interest. Log rank p-value is corrected for multiple testing using the Bon Ferroni method.

### Ethical approval

All animal experiments were approved by the King’s College London Animal Welfare and Ethical Review Board (AWERB) and the UK Home Office (Project licence P8DC5B496), in accordance with the relevant guidelines and practises. For studies using human tissue and clinical information, all methods were carried out in accordance with relevant guidelines and regulations, all experimental protocols were approved by a named institutional and/or licensing committee and informed consent was obtained from all participants or their legal guardians. For the Newcastle cohort, tumour samples were provided by the UK CCLG as part of CCLG-approved biological study BS-2007–04. Tumour investigations were done with approval from Newcastle North Tyneside Research Ethics Committee (study reference 07/Q0905/71). For the Toronto cohort, medulloblastoma samples were collected at diagnosis after obtaining informed consent from subjects as part of the Medulloblastoma Advanced Genomics International Consortium. Approval was obtained from institutional research ethics boards at all contributing institutions as outlined by Cavalli *et al*.^6^. For the Heidelberg cohort, all tumors were collected after receiving informed consent according to ICGC guidelines and approved by the institutional review board of contributing centers.

## Supplementary information

Supplementary information.
