# Supplementary material for: The AHR pathway represses TGFβ-SMAD3 signalling and has a potent tumour suppressive role in SHH medulloblastoma
Source: Sci Rep. 2020 Jan 10;10:148. doi: 10.1038/s41598-019-56876-z (PMC6954114; doi:10.1038/s41598-019-56876-z)

Supplementary information

**The AHR pathway represses TGFβ-SMAD3 signalling and has a potent tumour suppressive role in SHH medulloblastoma**

Nemanja Sarić^1^, Matthew Selby^2^, Vijay Ramaswamy^3^, Marcel Kool^4^, Brigitta Stockinger^5^, Christer Hogstrand^6^, Daniel Williamson^2^, Silvia Marino^7^, Michael D. Taylor^3^, Steven C. Clifford^2^ & M. Albert Basson^1, 8*^.

^1^Centre for Craniofacial and Regenerative Biology, King’s College London, Floor 27, Guy’s Hospital Tower Wing, London, SE1 9RT, UK.

^2^Wolfson Childhood Cancer Research Centre, Northern Institute for Cancer Research, Newcastle University, Newcastle-upon-Tyne, NE1 7RU, UK.

^3^Divisions of Hematology/Oncology and Neurosurgery, The Hospital for Sick Children, Toronto, ON, Canada. Departments of Medical Biophysics and Paediatrics, University of Toronto, Toronto, ON.

^4^Hopp Children´s Cancer Center (KiTZ), Division of Pediatric Neurooncology, German Cancer Research Center (DKFZ), and German Cancer Consortium (DKTK), Heidelberg, Germany.

^5^The Francis Crick Institute, 1 Midland Road, London, NW1 1AT, UK.

^6^Diabetes & Nutritional Sciences Division, King's College London, 3.85 Franklin-Wilkins Building, London, SE1 9NH, UK.

^7^Blizard Institute, Barts and The London School of Medicine and Dentistry, Queen Mary University of London, 4 Newark Street, London E1 2AT, UK.

^8^MRC Centre for Neurodevelopmental Disorders, King’s College London, 4^th^ floor, New Hunt’s House, London, SE1 1UL, UK.

**Figure S1. Conditional *Ahr* deletion from the GCP lineage results in reduced proliferation and enhanced cell cycle exit in the EGL.** (a) Method used to estimate quiescent fraction of GCPs in vivo. An initial IdU pulse followed by subsequent BrdU pulses were were given intraperitoneally to P7 control and *Ahr* cKO littermates, spaced 3 hours between each other, ensuring the initial fraction of IdU labelled cells had gone through the entire estimated GCP cell cycle length and had the opportunity to leave the cell cycle. (b) Staining of cerebellar vermis sections with antibody recognizing phosphorylated histone H3B (red) and counterstaining with Hoechst (blue). (c) Staining of vermis sections of IdU/BrdU pulsed control and *Ahr* cKO cerebella with antibodies recognizing both BrdU and IdU (green) or BrdU alone (red) and counterstaining with Hoechst (blue). IdU+ (N_Q_) and IdU+/BrdU+ (N_P+Q_) GCPs were counted from 2 separate bins/lobule (bin=100x100um) and used to estimate the Q fraction according to formula in (a). (d) Quantification of pH3B+ cells/lobule. Cell counts were normalized to lobule length. (e) Quantification of Q fraction/lobule. 3 non-adjacent sections from 3 cerebella of each genotype were used to obtain data in (d) and (e). Note significant reduction in proliferation and increase in cell cycle exit in anterior lobules of *Ahr* cKO cerebella. Data was statistically analyzed by Student’s t test (p<0.01 (**), p<0.05 (*)). Bars represent mean values +/- SEM. EGL-external granular layer, ML-molecular layer, IGL-internal granular layer. Scale bars: 300um (b, c (left panels)), 100um (c (right panels)).


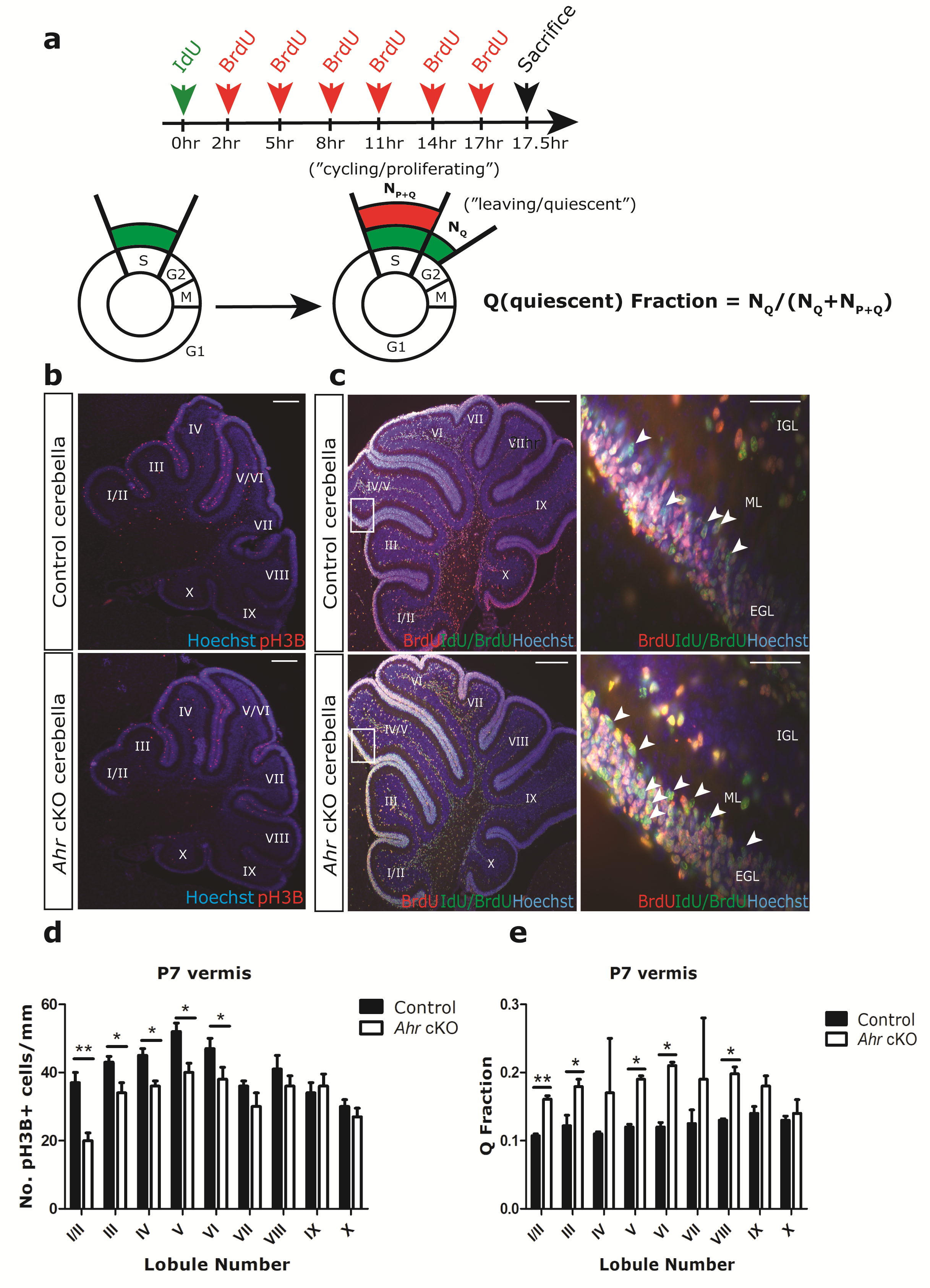


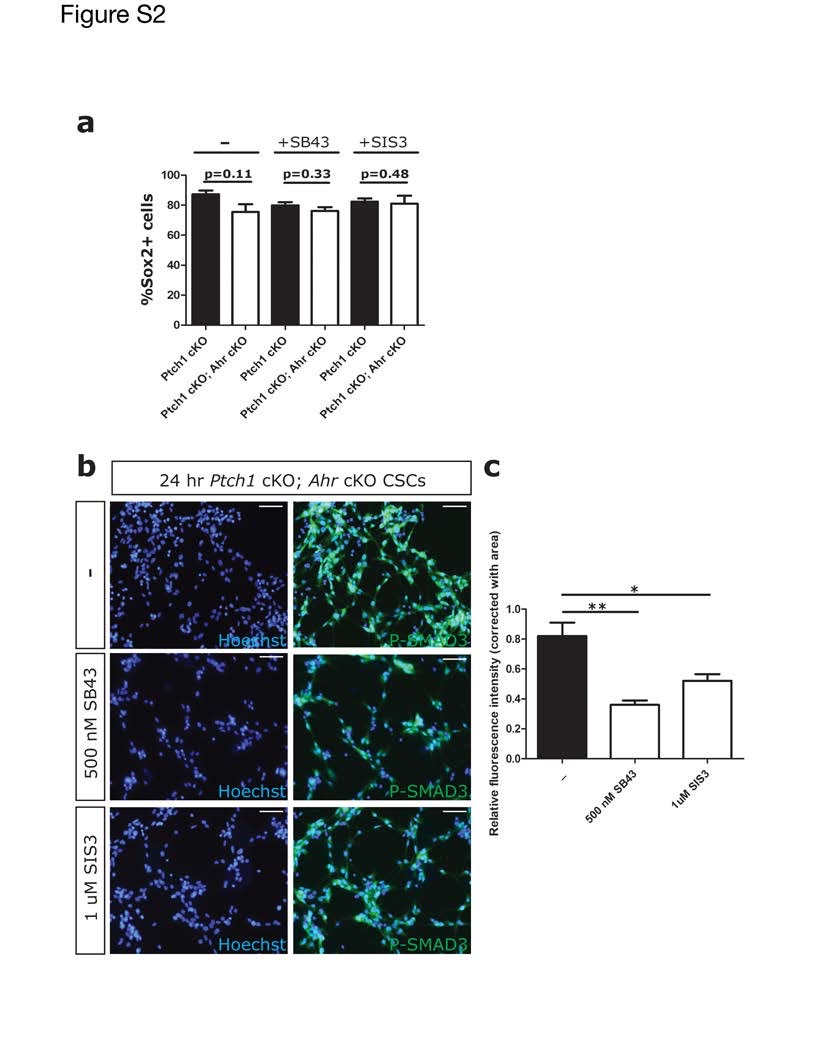
**Figure S2. Validation of TGF-β inhibitor doses in CPC cultures.** (a) Quantification of %Sox2+ cells in proliferating CPC cultures (Figure 5). On average 100 cells were counted from 4 different fields of view from triplicate wells of each condition. (b) Immunocytochemical staining for P-SMAD3 (green) in *Ptch1* cKO; *Ahr* cKO CPC cultures with/without added 500 nM SB-431542 (SB43) or 1 uM SIS3 for 24 hours. (c) Quantification of relative fluorescence intensity for cultures in (b). Note significant lowering of P-SMAD3 levels in inhibitor-treated cultures. Total cell count (Hoechst+ nuclei) data for (b) is given in Figure S4c Data was statistically analyzed by Student’s t test (p<0.01 (**), p<0.05 (*)). Bars represent mean values +/- SEM. Scale bars: 10um (b).


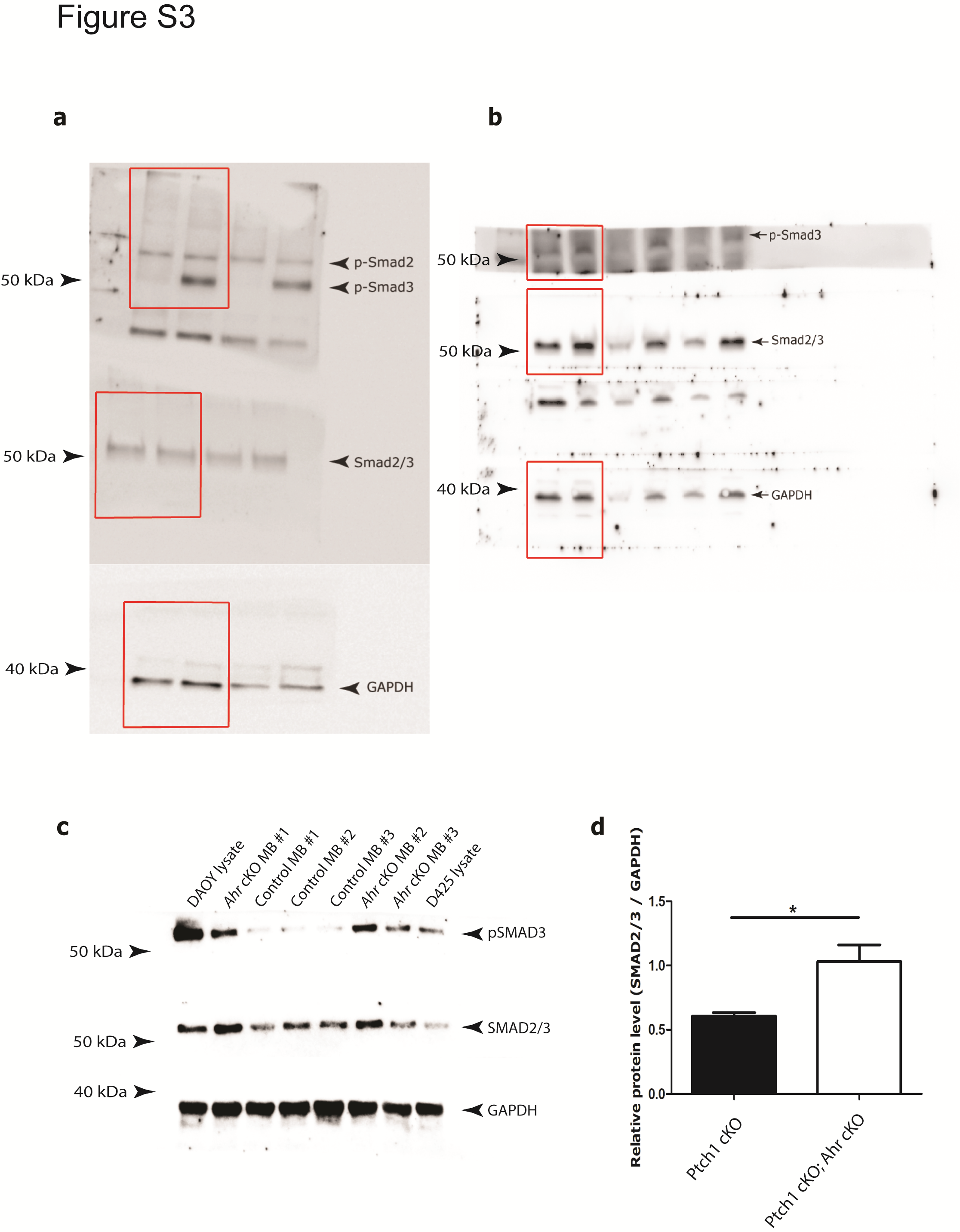


**Figure S3. Uncropped blot images.** (a) Figure 2a uncropped western blot. Red boxed areas show lanes cropped for final figure. (b) Figure 4a uncropped western blot. Red boxed areas show lanes cropped for final figure. (c) Full western blot of total cell lysates of isolated end stage medulloblastoma tissue with phosphorylated SMAD3 (pSMAD3), total SMAD2/3 and GAPDH proteins. (d) Quantification of band optic density for SMAD2/3 normalized to GAPDH levels. Data is representative of 3 animals/genotype.

**Figure S4. Total cell counts in figures 2 and 5.** (a) Quantification of total GCP number (Hoechst + nuclei) in each field of view of each condition (refers to Figure 2). (b) Quantification of total cell number (Hoechst+ nuclei) in each field of view of each condition (refers to Figure S2b).


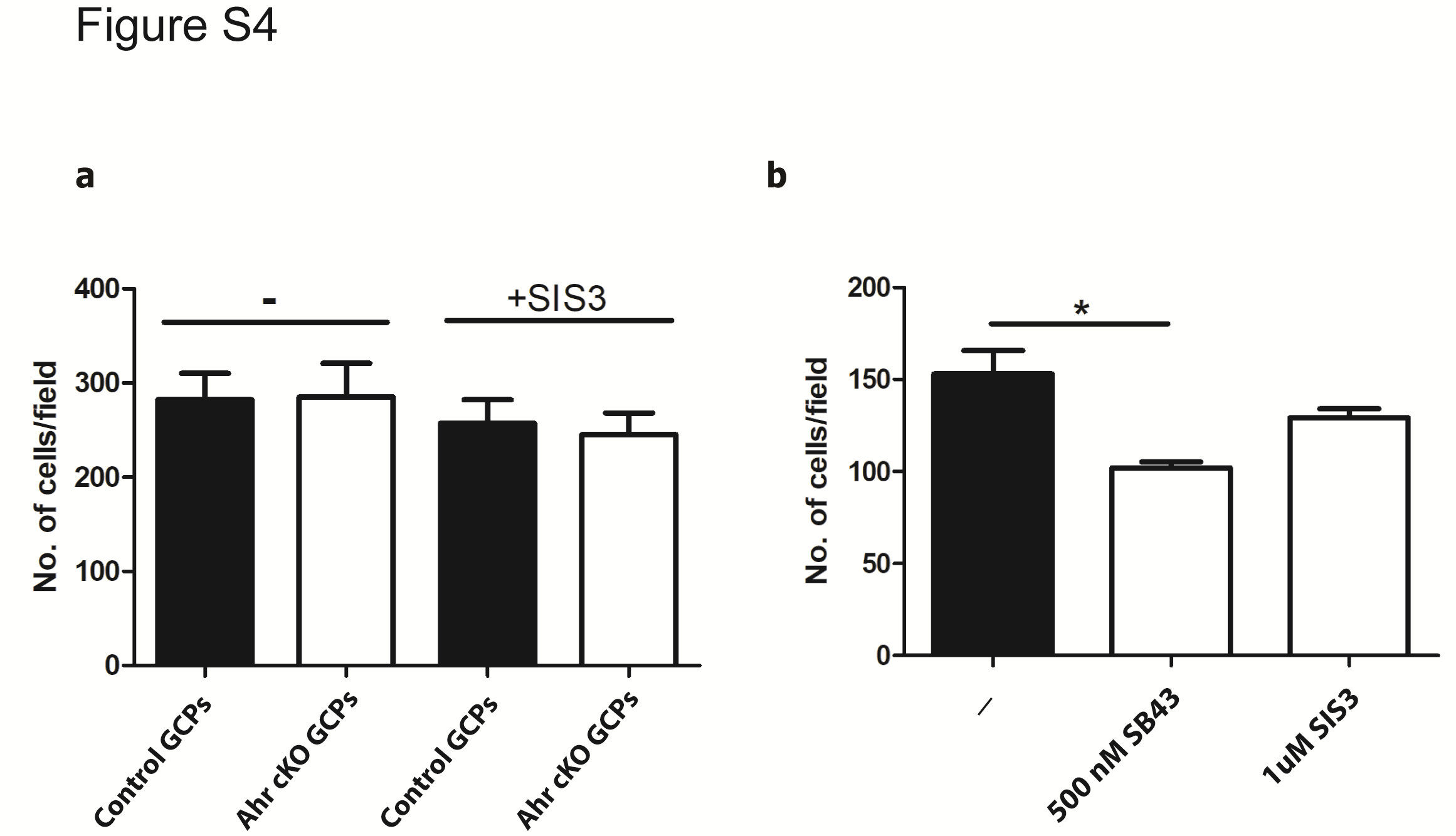

Supplement: Supplementary file 1 — Supplementary information. [file 41598_2019_56876_MOESM1_ESM.docx]
